# Oncolytic vaccinia therapy of squamous cell carcinoma

**DOI:** 10.1186/1476-4598-8-45

**Published:** 2009-07-06

**Authors:** Zhenkun Yu, Sen Li, Peter Brader, Nanhai Chen, Yong A Yu, Qian Zhang, Aladar A Szalay, Yuman Fong, Richard J Wong

**Affiliations:** 1 Department of Surgery, Memorial Sloan-Kettering Cancer Center, New York, NY, USA; 2 Beijing Tong Ren Hospital, Capital Medical University, Key Laboratory of Otolaryngology Head and Neck Surgery, Ministry of Education, PR China; 3 Department of Radiology, Memorial Sloan-Kettering Cancer Center, New York, NY, USA; 4 Genelux Corporation, San Diego Science Center, San Diego, California, USA; 5 Rudolf Virchow Center for Experimental Biomedicine, Institute for Biochemistry and Institute for Molecular Infection Biology, University of Würzburg, Am Hubland, D-97074, Würzburg, Germany

## Abstract

**Background:**

Novel therapies are necessary to improve outcomes for patients with squamous cell carcinomas (SCC) of the head and neck. Historically, vaccinia virus was administered widely to humans as a vaccine and led to the eradication of smallpox. We examined the therapeutic effects of an attenuated, replication-competent vaccinia virus (GLV-1h68) as an oncolytic agent against a panel of six human head and neck SCC cell lines.

**Results:**

All six cell lines supported viral transgene expression (β-galactosidase, green fluorescent protein, and luciferase) as early as 6 hours after viral exposure. Efficient transgene expression and viral replication (>150-fold titer increase over 72 hrs) were observed in four of the cell lines. At a multiplicity of infection (MOI) of 1, GLV-1h68 was highly cytotoxic to the four cell lines, resulting in ≥ 90% cytotoxicity over 6 days, and the remaining two cell lines exhibited >45% cytotoxicity. Even at a very low MOI of 0.01, three cell lines still demonstrated >60% cell death over 6 days. A single injection of GLV-1h68 (5 × 10^6 ^pfu) intratumorally into MSKQLL2 xenografts in mice exhibited localized intratumoral luciferase activity peaking at days 2–4, with gradual resolution over 10 days and no evidence of spread to normal organs. Treated animals exhibited near-complete tumor regression over a 24-day period without any observed toxicity, while control animals demonstrated rapid tumor progression.

**Conclusion:**

These results demonstrate significant oncolytic efficacy by an attenuated vaccinia virus for infecting and lysing head and neck SCC both *in vitro *and *in vivo*, and support its continued investigation in future clinical trials.

## Background

It is estimated that over 35,000 new cases of squamous cell carcinoma involving the oral cavity or pharynx were diagnosed in the United States in 2008, and that these malignancies led to over 7,500 deaths that year [1]. Despite refinements in the application of conventional cancer therapy modalities including radiation, chemotherapy, and surgery, the overall survival of patients with advanced head and neck squamous carcinoma remains limited [2]. Novel therapeutic agents are needed to improve poor outcomes for patients with head and neck cancer who fail conventional therapy.

Oncolytic viral therapy is a promising approach to cancer treatment that relies on the natural ability of viruses to infect, replicate within, and ultimately lyse a host cell. A variety of viruses have been demonstrated to exhibit oncolytic properties including adenovirus, herpes simplex virus, Newcastle disease virus, vesicular stomatitis virus, reovirus, among others [3]. Vaccinia has many favorable characteristics as an oncolytic virus, including the ability to infect a wide range of hosts, efficient infection and gene expression, and potent lytic activity [4]. Vaccinia viral replication occurs exclusively in the cytoplasm, which eliminates the possibility of chromosomal integration. One of the most attractive features of vaccinia as a therapeutic agent is its long and established safety record in its widespread historical use in humans as a vaccine for smallpox. Interestingly, vaccinia virus appears to naturally possess an intrinsic ability to selectively target and infect cancer cells *in vivo *[5]. The oncolytic activity of a recombinant, replication-competent vaccinia virus (GLV-1h68) in treating human thyroid cancer, breast cancer, malignant pleural mesothelioma, and pancreatic cancer was described before [6-10]. A GLV-1h68 derivative (GLV-1h99) that expresses the human nonepinephrine transporter was recently shown to be useful for both therapy and deep-tissue imaging of tumors [11,12]. In the current study, we sought to assess the utility of applying GLV-1h68 as a therapeutic agent against human head and neck squamous cell carcinoma both *in vitro *and *in vivo *using a murine flank tumor model.

## Results

### In vitro X-gal staining and GFP visualization

**Figure 1:**
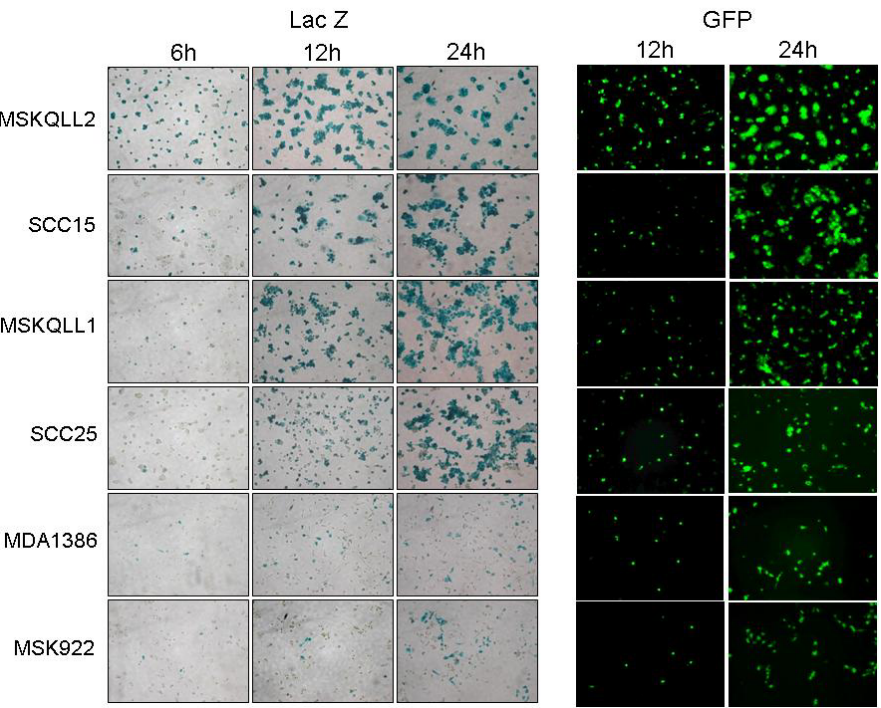
**Effective early infection by GLV-1h68 of human head and neck squamous cell carcinoma cell lines**. Six cell lines were infected by GLV-1h68 at an MOI of 1 and fixed in 1% glutaraldehyde for X-gal staining, or unfixed cells were visualized under a GFP filter. Photomicrographs were taken at 100×. All six cell lines showed progressively increasing marker gene expression within 24 hours, demonstrating effective infection at early time points.

X-gal histochemistry of six human HNSCC cell lines demonstrated evidence of infection by GLV-1h68 at 6 hours after exposure to GLV-1h68 at an MOI of 5 in all of the cell lines (Figure 1). Furthermore, there was intensely positive staining by four of the cell lines (MSKQLL2, SCC15, MSKQLL1, and SCC25) within 24 hours of exposure. The two other cell lines (MDA1386, MSK922) also demonstrated positive X-gal staining, although to a lesser degree. GFP assessment by microscopy showed peak expression at 24 hours and exhibited very similar findings as the X-gal staining, with the cell lines exhibiting GFP expression in the same relative order of intensity as demonstrated by the X-gal staining.

### In vitro quantification of β-galactosidase and luciferase enzymatic activities

**Figure 2:**
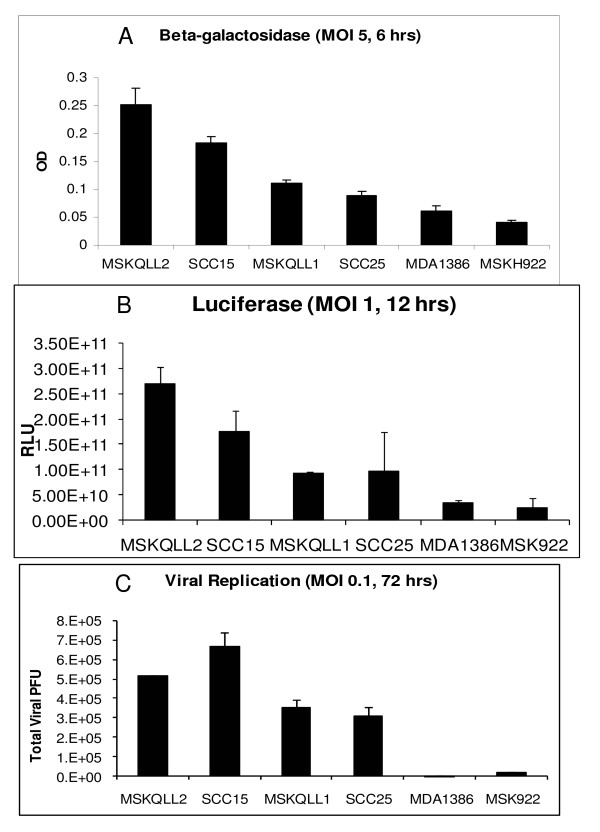
***In vitro *quantification of gene expression and viral replication by GLV-1h68 in human head and neck squamous cell carcinoma**. A. Cell lines were infected with GLV-1h68 at an MOI of 5, and six hours later quantitative β-galactosidase assays were performed. B. Cell lines were infected with GLV-1h68 at an MOI of 1, and 12 hours later coelenterazine was added. Bioluminescence was imaged with a cooled CCD camera and quantified with software analysis. C. Cell lines were exposed to GLV-1h68 at an MOI of 0.1 and incubated for 72 hours. Supernatants were collected and viral titers quantified by plaque assays on confluent CV-1 cells.

Quantitative assays for β-galactosidase expression by GLV-1h68 at an MOI of 5 reflected qualitative visual findings from X-gal staining, with MSKQLL2 showing the highest expression and MSK922 the lowest expression (Figure 2a). All of the cell lines demonstrated some degree of susceptibility to infection by GLV-1h68. Luciferase expression measured by software quantification of acquired photon emission images demonstrated very similar findings as the quantitative β-galactosidase assays, with the same relative order of expression noted (Figure 2b).

### Viral replication

Low dose GLV-1h68 at an MOI of 0.1 (2 × 10^3 ^pfu) was added to the cell lines for a three day course to allow for measurement of viral replication. The four HNSCC cell lines (MSKQLL2, SCC15, MSKQLL1, SCC25) most sensitive to viral infection as demonstrated by β-galactosidase and luciferase assays also supported robust viral proliferation, ranging from a >330-fold increase in GLV-1h68 titer (SCC15) to a >150-fold increase (SCC25) over the 3-day period (Figure 2c). In contrast, the less sensitive MSK922 and MDA1386 cell lines demonstrated a ten-fold increase and an unchanged viral titer, respectively.

### Cytotoxicity assays

**Figure 3:**
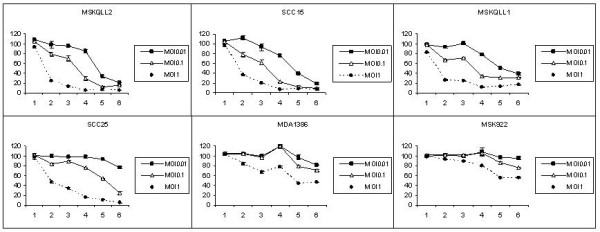
**GLV-1h68 exhibits significant oncolytic effects in human head and neck squamous cell carcinoma *in vitro***. Six cell lines were infected with GLV-1h68 at varying MOIs (0.01, 0.1, 1) and lactate dehydrogenase assays performed for 6 days to assess cell viability. Untreated cells were considered 100% viable. The y-axis designates percentage viability, and the x-axis designates the day post-infection. All six cell lines sustained significant cytotoxicity at an MOI of 1, four cell lines were sensitive at an MOI of 0.1, and three cell lines demonstrated an exquisite sensitivity to GLV-1h68 even at a very low MOI of 0.01.

GLV-1h68 incubated over a 6-day period with each of the six HNSCC cell lines demonstrated significant cytotoxicity against all cell lines at an MOI of 0.01, 0.1, and 1 (Figure 3). The relative cytotoxic sensitivities of the six cell lines reflected their relative susceptibility to GLV-1h68 infection by β-galactosidase and luciferase assessment. GLV-1h68 at an MOI of 1 was nearly completely cytotoxic by day 6 for the four most sensitive cell lines: MSKQLL2, SCC15, MSKQLL1, and SCC25. Even MDA1386 and MSK922 showed significant cytotoxicity to GLV-1h68 at MOI 1, with 47% and 55% viability by day 6, respectively. The lower MOI curves also reflect this same relative order of susceptibility. Even at a very low MOI of 0.01, MSKQLL2, SCC15, and MSKQLL1 demonstrated significant cell death by day 6, which reflects exquisite sensitivity by half of the cell lines treated with GLV-1h68.

### In vivo GLV-1h68 gene expression

**Figure 4:**
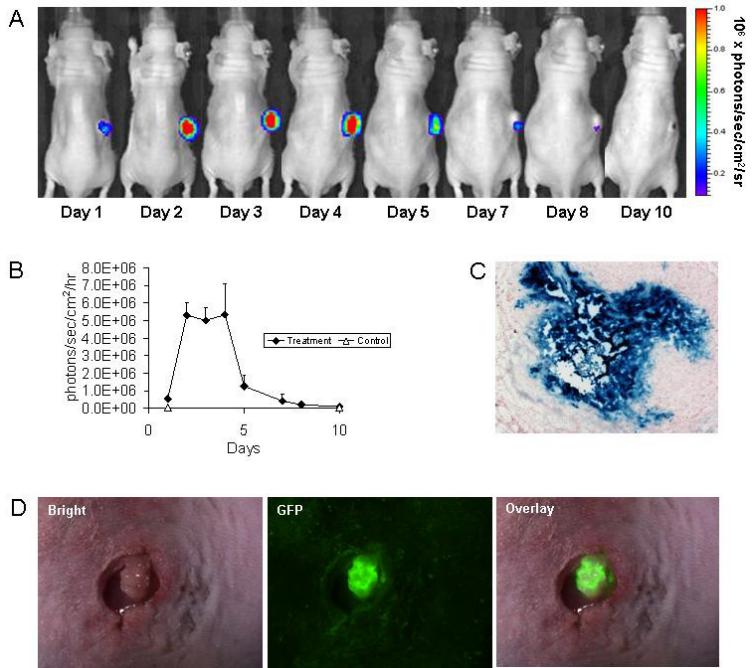
**GLV-1h68 imaging *in vivo *shows selective and prolonged virus-mediated luciferase activity in tumors**. A. Established MSKQLL2 flank tumors were injected with GLV-1h68 (5 × 10^6 ^pfu) and luciferase activity imaged following retro-orbital coelenterazine injections over a 10 day period. The late reduction of luciferase activity correlated with tumor size regression. B. Quantification of luciferase activity over a 10 day time course using software assessment of emitted photons was performed (n = 3 animals). C & D. MSKQLL2 flank tumors were excised 3 days after GLV-1h68 injection and stained for β-galactosidase expression (C) or examined under a GFP filter after tumors were exposed surgically (D) for photomicroscopy (100×).

Luciferase imaging demonstrated increasing activity remaining localized specifically to the MSKQLL2 flank tumor after the GLV-1h68 injection, with peak levels noted at days 2–4 (Figure 4a). There was a subsequent decline of luciferase activity to day 10. No luciferase activity was detected in other sites of the animal. At day 3, both β-galactosidase expression measured by X-gal staining (Figure 4c) and GFP expression (Figure 4d) appeared intense, reflecting effective viral gene expression.

### Therapy of human SCC xenografts with GLV-1h68 in mice

**Figure 5:**
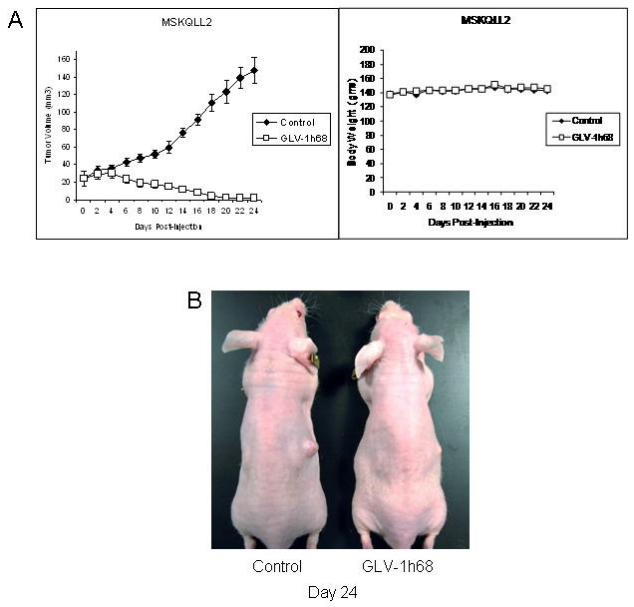
**A single injection of GLV-1h68 results in complete regression of established MSKQLL2 flank tumors *in vivo***. A. Established MSKQLL2 flank tumors (n = 5 per group) were treated with a single intratumoral injection of GLV-1h68 (5 × 10^6 ^pfu) and followed. Measured tumor volumes for the GLV-1h68 group demonstrated nearly complete regression, with three out of five animals showing complete tumor resolution and the other two demonstrating only a tiny 1–2 mm remnant of skin thickening at the regressed tumor site. In contrast, there was significant progression of tumor volume for the control, PBS-treated group. There was no observed change in animal weight related to viral administration, and no evidence of any toxicity. B. A representative animal from each group was photographed at day 24.

Established MSKQLL2 flank tumors with a mean starting volume of 24 mm^3 ^were treated with a single dose of intratumoral GLV-1h68 and the animals were followed for a period of 24 days. Treated tumors demonstrated progressive tumor volume regression over a three week period, and by day 24 the mean tumor volume was just 1.3 ± 0.9 mm^3 ^(Figure 5a). Three out of five animals had a complete regression of tumor, with the other two animals demonstrating only a tiny 1–2 mm remnant of skin thickening at the regressed tumor site. In contrast, all of the control tumors progressively increased in size to a mean volume of 148 ± 15 mm^3 ^over the same period (p < 0.001, t-test). Mean body weights remained stable for both control and GLV-1h68 groups (Figure 5a). There was no morbidity attributable to GLV-1h68 therapy identified in any animal. The experiment was concluded at day 24 due to ulceration of one of the control tumors.

## Discussion

Novel therapies are needed to make improvements in outcomes for patients afflicted with head and neck squamous cell carcinomas involving the upper aerodigestive tract [2]. Agents with novel mechanisms of anti-tumoral activity would be particularly useful for the treatment of recurrent cancers that have developed resistance to radiation therapy or chemotherapy. Oncolytic viral therapy is a new approach to cancer treatment that harnesses a virus's natural ability to infect, replicate within, and lyse a host cell as part of its natural life cycle [3,4].

Interestingly, there is compelling evidence that vaccinia virus may possess a natural affinity for infecting malignant tumors. Yu *et al *demonstrated that LIVP strain-derived vaccinia virus expressing *Renilla *luciferase and GFP was able to specifically infect C6 glioma, MB-49 murine bladder, and MCF-7 mammary neoplasms at 3 to 6 days after intravenous administration in mice [5]. Imaging of GFP expression demonstrated localization of virus at neoplastic sites measuring as small as 0.5 mm. In contrast, intravenous vaccinia administered to non-tumor-bearing animals only showed weak and occasional uptake in small skin lesions, without evidence of significant internal fluorescence localization.

GLV-1h68 is an attenuated, replication-competent, oncolytic vaccinia virus. GLV-1h68 has a mutation in the *J2R *gene encoding thymidine kinase which may make viral replication dependent on host cell thymidine kinase [6]. This attribute may create increased selectivity for malignant tumors but attenuate its ability to replicate in normal tissues. GLV-1h68 also carries an insertional mutation in hemagglutinin gene and the F14.5L locus. Inactivation of hemagglutinin is thought to reduce the virulence of vaccinia. F14.5L has a unique Not I site in F14.5L makes this locus suitable for insertion of the luciferase-GFP cassette. Lin *et al *showed that GLV-1h68 injections into established anaplastic thyroid tumors in murine flanks resulted in specific and prolonged luciferase expression within tumor tissues, while this was absent when GLV-1h68 was injected into normal tissue sites [8].

Our goal was to explore the potential utility of applying GLV-1h68 as a therapeutic agent for human head and neck squamous cell carcinoma. We examined the effects of GLV-1h68 in treating a panel of six human head and neck SCC cell lines *in vitro*. These six cell lines are of mucosal origin from the upper aerodigestive tract, and are commonly used to represent this disease. All six cell lines supported viral transgene expression (β-galactosidase, green fluorescent protein, luciferase) beginning as early as 6 hours after viral exposure. Highly efficient transgene expression and viral replication was observed in four of the six cell lines, suggesting that there are cancer cell factors that are determinants of permissiveness to vaccinia. The low dose of GLV-1h68 used in the viral replication studies was intended to allow assessment of maximal viral replication, and may have been insufficient to allow for initial infection of the less sensitive cell lines.

At an MOI of 1, GLV-1h68 was highly cytotoxic to four of the cell lines, resulting in ≥ 90% cytotoxicity over a 6-day period. Even at an extremely low MOI of 0.01, three cell lines still demonstrated > 60% cell death over 6 days. Therefore, half of SCC cell lines tested exhibited exquisite sensitivity to GLV-1h68. The other three SCC cell lines remained susceptible to oncolysis at higher doses of GLV-1h68, demonstrating that the virus may still exhibit efficacy at higher doses.

We found that markers of early gene expression by GLV-1h68 correlated well with susceptibility to oncolysis. β-galactosidase, GFP, and luciferase expression were all robust for MSKQLL2, SCC15, MSKQLL2, and SCC25; these were also the same cell lines that were also most sensitive to viral replication and oncolysis. These correlations suggest that initial viral entry and early gene expression by GLV-1h68 are important events that may define subsequent cytotoxic susceptibility to the virus. Clinically, this observation also suggests that luciferase imaging to visually track early viral activity *in vivo *may potentially have prognostic utility in predicting later tumor response to the virus. Bioluminescence imaging allows for the non-invasive detection of viral distribution and viral activity of GLV-1h68.

A single injection of intratumoral GLV-1h68 (5 × 10^6 ^pfu) *in vivo *into MSKQLL2 xenografts in mice exhibited localized intratumoral luciferase activity peaking at days 2–4, with gradual resolution over 10 days. Importantly, there was no evidence of viral spread to normal organs or non-tumor sites based on luciferase imaging, demonstrating tumor-specific viral replication and activity. Treated animals exhibited essentially complete tumor regression over a 24-day period without any observed toxicity, while control animals demonstrated rapid tumor progression requiring sacrifice by day 24. The lag period between the earlier resolution of GLV-1h68 luciferase activity and the later tumor volume regression might, in part, be explained by the additional time needed for the resorption of necrotic tumor tissue after effective oncolysis is completed. No animal treated with GLV-1h68 exhibited any morbidity attributable to the viral therapy. These results demonstrate that GLV-1h68 exhibits significant therapeutic efficacy leading to dramatic tumor regression without observed toxicity when delivered by intratumoral injection.

A major consideration in the development of a therapy using replication-competent viruses is the safety of its application in patients. Most oncolytic viruses such as adenovirus and herpes simplex virus are natural pathogens, and viral attenuation is necessary to make them safe for clinical use [3]. In contrast, the widespread historical use of vaccinia as a vaccine for smallpox has convincingly demonstrated its safety for clinical application in humans. Toxicities related to vaccinia administration occur in less than 0.1% of cases, and may be effectively addressed with immunoglobulin administration [13] and other possible antivirals such as cidofovir [14] and ST-246 [15]. The routine administration of vaccinia in the United States ended in 1972, and smallpox was officially declared eradicated in 1980. Therefore, the widespread historical clinical use of vaccinia virus in humans is strong evidence of its likely safety as a future cancer therapy agent. The additional genetic attenuation of GLV-1h68 may even further enhance its safety profile over wild type vaccinia virus. Our findings support the continued study of GLV-1h68 for treating head and neck squamous cell carcinoma in future clinical trials.

## Conclusion

These results highlight the ability of GLV-1h68 to effectively infect, replicate within, and cause regression of human head and neck squamous cell carcinoma xenografts in mice. No treatment related morbidity or mortality was identified. Vaccinia was historically used widely as a smallpox vaccine with an excellent safety profile. GLV-1h68 is even further attenuated for safety, while maintaining potent antitumor activity. These data support the continued study of GLV-1h68 as an agent to treat patients with head and neck squamous cell carcinoma in future clinical trials.

## Methods

### Virus and cell lines

GLV-1h68 is a recombinant, replication-competent vaccinia virus derived from the LIVP strain (Lister strain from the Institute for Research on Viral Preparations, Moscow). The construction of GLV-1h68 has been described before [5]. GLV-1h68 contains four inserted cassettes encoding *Renilla *luciferase-green fluorescent protein fusion (*RUC*-*GFP *cassette), a reverse inserted human transferrin receptor (*rTfr*), β-galactosidase, and β-glucuronidase into the *F14.5L*, *J2R *(thymidine kinase), and *A56R *(hemagglutinin) loci of the viral genome, respectively.

Six human head and neck squamous cell carcinoma cell lines were studied: MSKQLL2, SCC15, MSKQLL1, SCC25, MDA1386, and MSK922. MDA1386 was grown in RPMI containing 10% FCS and 1% penicillin and streptomycin (P&S), all other cell lines were grown in MEM containing 10% FCS and 1% P&S. CV-1 African green monkey kidney cells used for viral tittering were grown in DMEM containing 10% FCS and 1% P&S. All cells were grown in a humidified incubator at 5% CO_2 _and 37°C.

### In vitro X-gal staining and GFP visualization

Cells were seeded in 12 well plates for 6 hours and exposed to GLV-1h68 at an MOI of 1. At 6, 12, and 24 hours after virus infecton, cells were fixed in 1% glutaraldehyde and stained with X-Gal (1 mg/ml) in an iron solution of 5 mM K_4_Fe (CN)_6_, 5 mM K_3_Fe(CN)_6 _and 2 mM MgCl_2 _at 37°C for 2 hours. Photomicrographs were taken at 100 ×. At 12 and 24 hours, unfixed cells were imaged under a microscope with a GFP filter at 100 ×.

### In vitro quantification of β-galactosidase activity

β-galactosidase expression was measured using the Enhanced β-Galactosidase Assay Kit (Gene Therapy Systems, San Diego, CA). Each cell line was plated in 96-well plates at 2 × 10^4 ^cells per well in 100 μl DMEM with 2% FCS media for 6 hours. GLV-1h68 in 100 μl media was added to each well at an MOI of 5. After 6 hours, media was aspirated and lysis buffer added to each well. Serial dilutions of β-galactosidase were used to create a standard reference curve. Substrate was added to each well and plates read by spectrophotometry (EL321e, Bio-Tek Instruments, Winooski, VT) at 570 nm. Samples were assayed in triplicate for each condition and mean values with standard errors reported.

### In vitro quantification of luciferase activity

For luciferase assessment, cells were plated in 96-well plates at 1 × 10^4 ^cells per well and 6 hours later GLV-1h68 was added at an MOI of 1. 12 hours after infection, 0.25 μg coelenterazine (Biotium, Hayward, CA) in 50 μl PBS was added to each well for 10 minutes, and emitted photons were measured for 30 seconds with a cooled CCD camera (Xenogen IVIS, Xenogen, Alameda, CA). Images were analyzed by using Living Image^® ^software (Xenogen). Samples were assessed in triplicate.

### Viral plaque assays

To assess viral replication, 2 × 10^4 ^cells were seeded per well in 12-well plates in 1 ml media. After incubation for 6 hours, GLV-1h68 in 100 μl DMEM 2% FCS was added to each well at an MOI of 0.1 (2 × 10^3 ^pfu). Supernatant from each well was collected at 72 hours and frozen. CV-1 cells were grown to confluence on 6-well plates. Supernatant samples were thawed, and serial ten-fold dilutions incubated on the CV-1 cells for 4 hours. Wells were washed with media and covered with 1% agarose with media. After 48 hours of incubation, 2 mL of neutral red solution (2% by volume) was added and viral plaques were counted after 24 hours. Samples were assayed in triplicate for each condition and mean values with standard errors reported.

### Cytotoxicity assays

Each cell line was plated in 12-well plates. After incubation for 6 hours, virus in 100 μl DMEM with 2% FCS media was added to each well at an MOI of 0, 0.01, 0.1 and 1. After day 3, an additional 1 ml of fresh media was added to remaining wells. At daily intervals cells were washed with PBS and lysed with Triton X (1.35%, Sigma). Lactate dehydrogenase (LDH) was measured using a Cytotox96 kit (Promega, Madison, WI) and measured by spectrophotometry (EL321e, Bio-Tek Instruments, Winooski, VT) at 450 nm. Results were expressed as the percentage of surviving cells determined from comparing the lactate dehydrogenase of each test sample to untreated samples considered 100% viable. Samples were assayed in triplicate for each condition and mean values with standard errors reported.

### In vivo GLV-1h68 gene expression

*In vivo *experiments were performed under an approved animal protocol by the Memorial Sloan-Kettering Institutional Animal Care and Use Committee. Six-week-old male athymic nude mice (National Cancer Institute) were anesthetized with inhalational methoxyflurane. MSKQLL2 cells (5 × 10^6^) in 100 μl of PBS were injected into the subcutaneous flanks. Established MSKQLL2 flank tumors were injected with GLV-1h68 (5 × 10^6 ^pfu) in 50 μl PBS. For luciferase assessment, 2.5 μg of coelenterazine (Biotium) in 95 μl PBS was injected via the retro-orbital sinus at varying times. Luciferase activity was detected with a cooled CCD camera (Xenogen IVIS). Emitted photons were measured for 60 seconds. Images were analyzed using Living Image^® ^software (Xenogen).

For X-gal staining, at day 3 animals were sacrificed and flank tumors excised, frozen in Tissue Tek solution and sectioned (8 μm). Slides were fixed with 1% glutaraldehyde and stained with X-Gal (1 mg/ml) in an iron solution of 5 mM K_4_Fe (CN)_6_, 5 mM K_3_Fe(CN)_6 _and 2 mM MgCl_2 _at 37°C for 2 hours. Slides were counterstained with nuclear fast red. For GFP assessment, animals were sacrificed at day 3, tumors exposed surgically, and photography performed under a stereoscope (Leica MZFL3) with a GFP filter.

### Therapy of human SCC xenografts with GLV-1h68 in mice

Animals with established MSKQLL2 flank tumors were distributed into two experimental groups (n = 5 per group) with similar starting tumor volumes. Flank tumors were treated with intratumoral injections of a single dose of GLV-1h68 (5 × 10^6 ^pfu) in 50 μl PBS, or 50 μl PBS alone as control. Tumor dimensions were serially measured with calipers and volumes calculated by the formula: volume = length × width^2 ^× 0.4. Body weights were measured serially. Photographs of representative animals were taken at day 24. Animals were sacrificed by CO_2 _inhalation.

## Competing interests

ZY, SL, PB, YF, RJW declare that they have no competing interests. NC, YAY, QZ, AAS are employees of Genelux Corporation who receive salary and hold stock from Genelux Corporation. GLV-1h68 has been patented.

## Authors' contributions

ZY designed and performed all of the *in vitro *studies and analyzed the results. SL performed the *in vivo *studies. PB performed the luciferase imaging. NC, YAY, QZ, AAS produced GLV-1h68 and edited the manuscript. YF analyzed the results and edited the manuscript. RJW conceived and organized the study, analyzed the results, and drafted and edited the manuscript. All authors read and approved the final manuscript.
